# The Usefulness of Imaging Quantification in Discriminating Non-Calcified Pulmonary Hamartoma From Adenocarcinoma

**DOI:** 10.3389/fonc.2020.568069

**Published:** 2020-10-22

**Authors:** Xiaojun Guan, Shaoze Wang, Pingding Kuang, Haitong Lu, Minming Zhang, Dahong Qian, Xiaojun Xu

**Affiliations:** ^1^ Department of Radiology, The Second Affiliated Hospital, Zhejiang University School of Medicine, Hangzhou, China; ^2^ Institute of Very Large Scale Integrated-circuits (VLSI) Design, Zhejiang University, Hangzhou, China; ^3^ Institute of Translational Medicine, Zhejiang University School of Medicine, Hangzhou, China; ^4^ School of Biomedical Engineering, Shanghai Jiao Tong University, Shanghai, China

**Keywords:** imaging quantification, texture, non-calcified hamartoma, lung adenocarcinoma, radiomics

## Abstract

**Background:**

Patients with non-calcified hamartoma were more susceptible to surgery or needle biopsy for the tough discrimination from lung adenocarcinoma. Radiomics have the ability to quantify the lesion features and potentially improve disease diagnosis. Thus, this study aimed to discriminate non-calcified hamartoma from adenocarcinoma by employing imaging quantification and machine learning.

**Methods:**

Forty-two patients with non-calcified hamartoma and 49 patients with adenocarcinoma were retrospentation; Manual lesion segmentation, feature quantification (e.g., texture features), and artificial neural network were performed consecutively. Independent t-test was used to conduct the inter-group comparisons of those imaging features. Receiver operating characteristic curve was performed to investigate the discriminating efficacy.

**Results:**

Significantly higher contrast, cluster prominence, cluster shade, dissimilarity, energy, and entropy in non-calcified hamartoma were observed compared with lung adenocarcinoma. Texture-grey-level co-occurrence matrix showed a well discrimination between non-calcified hamartoma and adenocarcinoma as the detection sensitivity, specificity, accuracy, and the area under the curve were 87.22% ± 9.07%, 82.64% ± 8.07%, 85.11% ± 5.40%, and 0.942, respectively.

**Conclusion:**

Quantifying imaging features is a potentially useful tool for clinical diagnosis. This study demonstrated that non-calcified hamartoma has a heterogeneous distribution of attenuations probably resulting from its complex organizations. Based on this property, imaging quantification could improve discrimination of non-calcified hamartoma from adenocarcinoma.

## Introduction

Pulmonary hamartoma is the most common benign tumor in the lung, which constitutes approximately 8% of all neoplasms (1, 2). Patients with pulmonary hamartoma require no further treatment unless the lesion grows rapidly or the patients become symptomatic during clinical follow-up (2); only clinical monitoring is required for some patients with confirmed symptoms (3). The presences of fat and calcification in computed tomography (CT) detection have been reported to be good indicators of pulmonary hamartoma (2), and nearly 45% of 89 hamartomas were diagnosed depending on needle biopsy (1). However, about 35% of hamartomas lack of the appearance of fat or calcification (2), and the finding of fat was with little specificity in discriminating benign from malignant tumors (4). In a clinical practice, Cicco et al. (5) reported that only 26/42 (62%) of hamartoma patients in CT examination (calcification was found in 9 patients) were diagnosed as probably benign. Alternatively, positron emission tomography examination reached an accuracy of 81%, indicating that nearly 20% of the patients still had uptake characteristics suggesting malignancy (5). Therefore, for diagnostic and therapeutic purposes, these patients with non-calcified hamartoma (NCH) were susceptible to suffer from unneeded surgery or needle biopsy.

Different solid tumors have different biological bases varying from the density of tumor proliferation and the tissue components, and by taking the advantages of quantitative imaging technology this intra-tumoral heterogeneity can be reflected by calculating the complicated distributions of CT attenuations, termed imaging heterogeneity (6, 7). Therefore, radiomics, possessing the ability to quantify the high-dimension mineable features and identify the underlying differences, appears to offer a nearly limitless supply of imaging biomarkers that could potentially improve disease diagnosis (6–9). Specifically, texture-based features have been widely applied in the recognition tasks of pulmonary nodule, which could provide quantitative interrelationships between voxels and therefore capture the intra-tumoral heterogeneity (10–13). Taken together, we hypothesized that quantifying the high-dimension imaging features, especially the texture-based ones, would contribute to their discriminations and potentially reduce the invasive operations for patients with NCHs.

In the present study, we retrospectively collected 42 patients with NCH and 49 patients with adenocarcinoma. Procedures, including manual segmentation of lesions, feature extraction, and artificial neural network (ANN), were performed. This study aimed to discriminate NCH from lung adenocarcinoma by using imaging quantification and determine the internal biological behavior within these two tumors.

## Materials and Methods

### Subjects

This retrospective study was approved by the Medical Ethic Committee in the Second Affiliated Hospital, Zhejiang University School of Medicine, with a waiver of patients’ approvals. We retrospectively collected 42 patients with NCH (female/male, 22/20) in the past five years. All of them showed solid lesion, with the largest diameters from 6.6 to 32.4 mm, and accepted either thoracic surgery or needle biopsy. No one was diagnosed with a calcification (density > 120 HU) by experienced radiologists in our institute. Considering the distribution of these lesions, we found 6 lesions in the right superior lobe, 17 lesions in the right inferior lobe, 10 lesions in the left superior lobe, and 9 lesions in the left inferior lobe.

Forty-nine patients (female/male, 34/15) diagnosed as solid adenocarcinoma (the largest diameters from 9.1 to 40.0 mm) were also identified by the pathologists in the same institute. The lesions that had obvious cavitation and vessels passing through were excluded. The biggest lesion confirmed by pathology was segmented in a patient with pulmonary metastasis. All of these adenocarcinoma lesions were confirmed as having no calcification inside. To exclude the influence of obstructive pneumonia on feature extraction, the same radiologists confirmed that all lesions were solitary without obvious obstructive pneumonia. In addition, there were 11 lesions in the right superior lobe, 6 lesions in the right middle lobe, 14 lesions in the right inferior lobe, 7 lesions in the left superior lobe, and 11 lesions in the left inferior lobe.

**Table 1** showed the demographic information and lesion descriptions.

**Table 1:** Demographic information and lesion descriptions.

	Non-calcified Hamartoma	Lung Adenocarcinoma	*p* value
Number (Female/Male)	42 (22/20)	49 (34/15)	0.096
Age (years)	55.2 ± 11.4	56.8 ± 12.0	0.413
Location			0.092
Right superior lobe	6	11	
Right middle lobe	0	6	
Right inferior lobe	17	14	
Left superior lobe	10	7	
Left inferior lobe	9	11	
Diameter (mm)	14.3 ± 6.0	22.6 ± 7.4	<0.05

### Data Collection

The thorax images were obtained from four CT scanners in the institute (Siemens Sensation 16-detector, Siemens Volum Zoom 4-detector, Siemens Definition AS 32-detector, GE Right Seep RT 16-detector) with a breath-held helical acquisition, 120 or 140 kV, 120–240 mAs, and pitch 1.0–1.45. The collimations of them were 0.75, 0.625, 0.625, and 0.6 mm, respectively. All chest images were reconstructed with a reconstruction algorithm. The reconstructed slice thicknesses were 1.2–1.5 mm and the FOVs were 318–378 mm with a matrix 512 × 512 mm.

### Imaging Quantification

**Figure 1:**
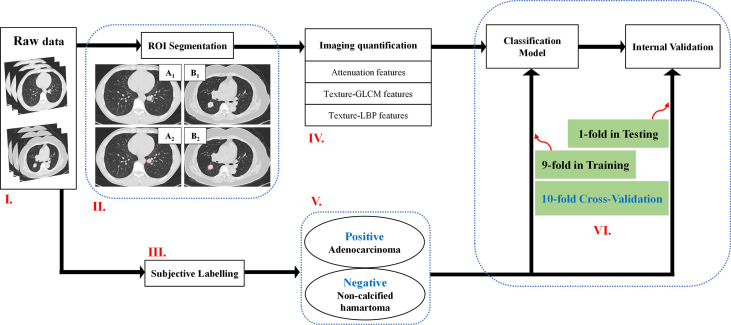
A flowchart of the imaging quantification. Flowchart I: Raw data. Flowchart II: ROI segmentation. Pathologically confirmed non-calcified hamartoma from a male patient with 44 years old locating in the left inferior lobe (A_1_–A_2_), pathologically confirmed adenocarcinoma from a female patient with 70 years old located in the right inferior lobe (B_1_–B_2_). Flowchart III and V: Adenocarcinoma patients were labelled as positive group; Non-calcified hamartoma patients were labelled as negative group. Flowchart IV and VI: Three kinds of features, e.g., attenuation features, GLCM features, and LBP features, were respectively extracted, and were trained by ANN model with 10-flod cross-validation method. ROI, Region of interest; ANN, Artificial neuronal network; GLCM, Gray-level co-occurrence matrix.

Three-dimensional (3D) ROIs were manually extracted using MITK open-source software in a slice-by-slice method. Two experienced radiologists (XG and XX) who were blind to the patient classifications conducted the 3D ROI segmentation (**Figure 1**). These ROI masks, stored in NIFT format, were converted into a ROI list where axial positions (x and y), slice indices (z), and CT attenuation values of each voxels were recorded. Attenuation values were normalized between μ ± 3σ where μ denotes the mean value and σ the standard deviation. This normalization is to reduce inter-scanner effects in CT feature analysis. Both 3D and two-dimensional (2D) features were extracted (**Table 2**): 3D attenuation features included mass, sigmoid function parameter, and statistical attenuation; 2D texture features included gray-level co-occurrence matrix (GLCM) and local binary pattern (LBP). Attenuation features record global pixel distribution without considering neighborhood restraint, while both GLCM and LBP consider neighborhood restraint. Moreover, GLCM makes statistics on global neighborhood restraint, while LBP just accounts local neighborhood restraint (14–17). Of note, GLCM and LBP were computed from the maximum-area slice in each ROI.

**Table 2:** The information of 94 features in detail.

3D attenuation features	
Mass	f_1_: sum of ROI voxel intensities *divided by voxel numbers*
Sigmoid	Get sigmoid features f_2_, f_3_ and f_4_ from curve fitting function: $\text{f}(\text{x})=\frac{{\text{f}}_{2}}{1+{\text{e}}^{-\frac{\text{x}-{\text{f}}_{3}}{{\text{f}}_{4}}}}$
Attenuation	f_5_: mean value of attenuation f_6_: standard deviation of attenuation f_7_: skew value of attenuation f_8_: kurtosis value of attenuation
**2D texture features**	
GLCM features on four orientations (0°, 45°, 90°, 135°)	f_9,29,49,69_: auto-correlation^**^ f_10,30,50,70_: contrast^**^ f_11,31,51,71_: correlation^**^ f_12, 32,52,72_: cluster prominence^**^ f_13,33,53,73_: cluster shade^**^ f_14,34,54,74_: dissimilarity^**^ f_15,35,55,75_: energy^**^ f_16,36,56,76_: entropy^***^ f_17,37,57,77_: homogeneity^*^ f_18,38,58,78_: maximum probability^*^ f_19,39,59,79_: sum of squares/variance^**^ f_20,40,60,80_: sum average^*^ f_21,41,61,81_: sum variance^**^ f_22,42,62,82_: sum entropy^***^ f_23,43,63,83_: difference variance^**^ f_24,44,64,84_: difference entropy^***^ f_25,45,65,85_: information measure of correlation 1^**^ f_26,46,66,86_: information measure of correlation 2^**^ f_27,47,67,87_: normalized inverse difference^***^ f_28,48,68,88_: normalized inverse difference moment^***^
LBP	f_89_: mean value of LBP codes^*^ f_90_: standard deviation of LBP codes^**^ f_91_: skew of LBP codes^***^ f_92_: kurtosis of LBP codes^***^ f_93_: histogram mean value^*^ f_94_: entropy of LBP codes^***^

*indicates first-order texture feature;

**indicates second-order texture feature;

***indicates high-order texture feature.

For texture-GLCM, the normalized attenuation values were decimated to 8 gray levels, based on which four co-occurrence matrices were generated to represent texture distribution on 0°, 45°, 90°, and 135°, respectively. On each orientation, 20 features were selected according to the previous studies (**Table 2**) (14, 16, 17). For texture-LBP, we computed rotation-invariant LBP codes and calculated both spatial and histogram features of LBP codes (15).

**Figure 2:**
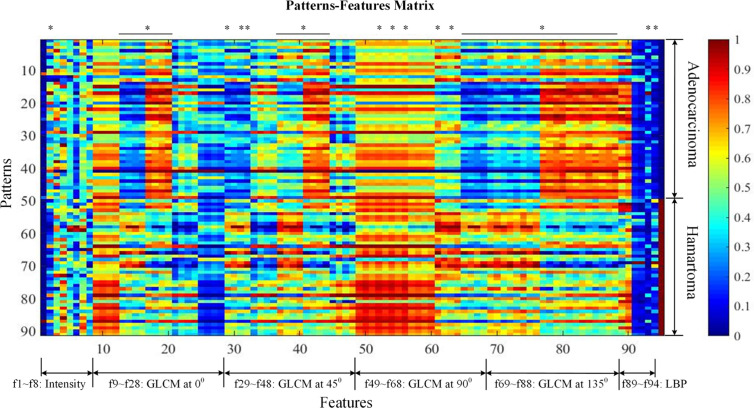
Patterns-features matrix (the quantification of 94 extracted features). Each row represents one sample recording 94 features (from left to right, mass feature, f1; sigmoid features, f2–f4; attenuation features; GLCM features at 0 degrees, f9–f28; GLCM features at 45 degrees, f29–f48; GLCM features at 90 degrees, f49–f68; GLCM features at 135 degrees, f69–f88; LBP features, f89–f94), and the target index on the last column (0 denotes adenocarcinoma and 1 non-calcified hamartoma). Prior to visualization, features on each column were linearly normalized to be within 0 and 1, respectively. * was considered as statistically significant after Bonferroni correction (p < 0.0005). GLCM, Gray-level co-occurrence matrix.

**Figure 2** visualized features as well as the classification targets of 91 subjects. Each row represented one sample, recording 94 features (from left to right, attenuation features, f1–f8; GLCM features at 0 degrees, f9–f28; GLCM features at 45 degrees, f29–f48; GLCM features at 90 degrees, f49–f68; GLCM features at 135 degrees, f69–f88; LBP features, f89–f94), and the target index on the last column (0 denoted adenocarcinoma and 1 NCH). Prior to visualization, features on each column were linearly normalized to be within 0 and 1, respectively.

ANN has been demonstrated to be more relevant to human brain perception and more flexible to be extended to deep learning classifiers such as deep stack networks or convolution neural networks (18). Therefore, in the present study, the ANN was implemented using Matlab feedforward networks that can be trained to classify the above features according to target labels. Here, a total of 94 features (80 GLCM features, 6 LBP features, and 8 attenuation features) were taken as input neurons. Since this paper aims to discriminate NCH from adenocarcinoma, two output neurons are adequate. The hidden neuron size was optimized to be 50 by 10-fold cross-validation; 100 training-testing cycles were conducted.

As a reference, this study invited two experienced radiologists (XX and LY, with 20 and 8 years of experience in diagnostic radiology) to review the CT images and make a clinical diagnosis according to their clinical knowledge. Both radiologists were unaware of clinical and pathologic results. Agreement would be achieved after a discussion if diagnostic inconsistency occurred.

### Statistical Analysis

Independent t-test was performed to test the intergroup difference of age distribution, and chi-square test was performed to analyze the differences of sex distribution and lesion location between patients with NCH and adenocarcinoma.

DICE similarity coefficient was performed to analyze the inter-observer variability in the lesion (ROI) segmentations between two radiologists, where the coefficient > 0.7 indicates an excellent agreement (19, 20). To compare the intergroup differences among 94 features, independent test was performed. We used Bonferroni correction to reduce the type I error, so that p < 0.0005 (0.050/94) was considered to be statistically significant. As 80 features of texture-GLCM were calculated on 4 orientations, to confirm its overall efficacy, features attaching to different orientations were averaged so that 20 pooled GLCM-based features were analyzed. The results were corrected by Bonferroni correction, and p < 0.0025 (0.050/20) was considered to be statistically significant.

The receiver operating characteristic curves (ROC) are plotted afterwards, and once the ROC curve is plotted, we can get sensitivity, specificity, accuracy, positive predictive value (PPV), negative predictive value (NPV), and the area under curve (AUC) between the positive group (NCH) and the negative (adenocarcinoma). Analyses of covariance (ANOVA) were used to compare the AUC distributions among different ANN models fed with texture-GLCM, texture-LBP, attenuation, and all features. Bonferroni method was used for the multiple comparison correction.

## Results

### Demographic and Clinical Statistic

A total of 91 patients were recruited in the present study, including 42 patients with NCH and 49 patients with adenocarcinoma (**Table 1**). There were no significant differences in the distributions of age (p = 0.413), sex (p = 0.096), and lesion location (p = 0.092) between patients with NCH and adenocarcinoma. Compared with the mean diameter of the adenocarcinoma (22.6 ± 7.4 mm), the NCH had a smaller mean diameter (14.3 ± 6.0 mm) (p < 0.050).

### Features Extraction

A total of 94 features, including attenuation, texture-GLCM, and texture-LBP, were extracted quantitatively from each lesion of NCH and adenocarcinoma (**Figure 2**). Intergroup comparisons showed that there were 53 features with significantly differences, 50 of which were derived by texture-GLCM features (p < 0.0005) (**Figure 2**).

**Figure 3:**
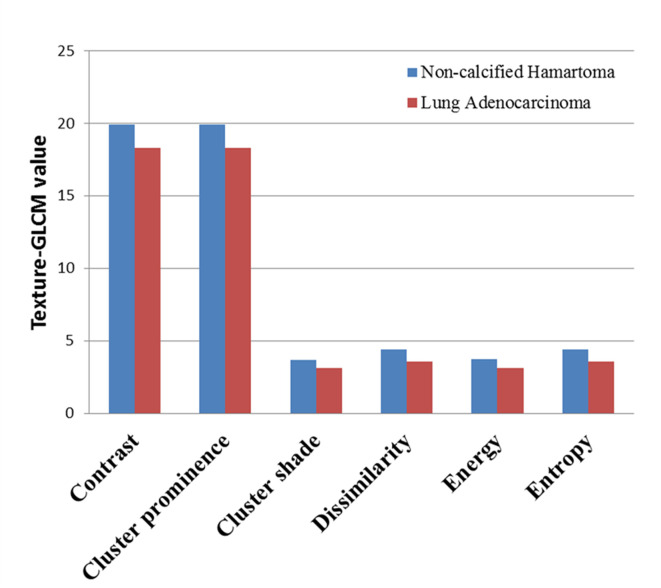
The averaged value of texture-GLCM features on 4 orientations that showed significant difference between non-calcified hamartoma and adenocarcinoma (after Bonferroni correction, p < 0.0025). GLCM, Gray-level co-occurrence matrix.

Of note, each feature defined by texture-GLCM was calculated on 4 different orientations (0°, 45°, 90°, and 135°). Therefore, we averaged each feature of texture-GLCM with 4 orientations to observe the overall differences between two groups. After Bonferroni correction, averaged features, such as contrast, cluster prominence, cluster shade, dissimilarity, energy, and entropy, in the NCH were significantly higher than that in the lung adenocarcinoma (P < 0.0025) (**Figure 3**).

### Discriminating Efficacy of Imaging Quantification

**Figure 4:**
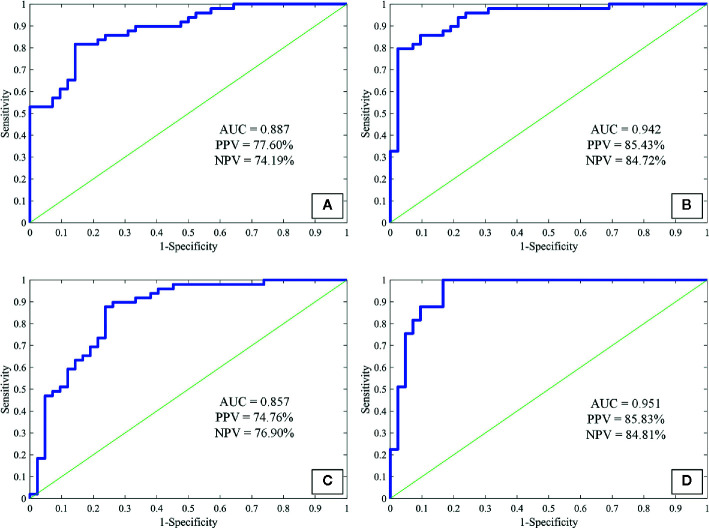
The receiver operating characteristic curves of attenuation features **(A)**, texture-GLCM **(B)**, texture-LBP **(C)**, and all features **(D)** to discriminate non-calcified hamartoma and adenocarcinoma. GLCM, Gray-level co-occurrence matrix; LBP, Local binary pattern; PPV, Positive predictive value; NPV, Negative predictive value.

As a preliminary study to explore the underlying clinical application of imaging quantification, we tested three commonly used feature extraction techniques in the present study. As texture-GLCM features derive from 4 isotropic sub-bands, i.e., 4 different orientations, averaging across all orientations, provided a perspective of statistical heterogeneous distribution from which lesions with diverse imaging attributes were projected into particular orientations. This is helpful for machine learning techniques to predict the target of a lesion comprehensively with low computing complexity. We observed that the sensitivity, specificity, accuracy, and AUC were 87.22% ± 9.07%, 82.64% ± 8.07%, 85.11% ± 5.40%, 0.942 respectively for the ANN model trained with texture-GLCM and 82.63% ± 11.38%, 67.46% ± 11.53%, 75.63% ± 7.09%, and 0.857 for that trained with texture-LBP. For the attenuation features, the sensitivity, specificity, accuracy, and AUC were 78.01% ± 10.55%, 73.73% ± 11.87%, 76.03% ± 7.58%, and 0.887, respectively. Finally, by combining the whole 94 features, the sensitivity, specificity, accuracy, and AUC were as follows: 87.23% ± 10.18%, 83.20% ± 8.61%, 85.37% ± 6.23%, and 0.951. **Figure 4** showed the performance of each ANN model fed with corresponding feature set; AUC, PPV, and NPV were exhibited.

Among the AUC distributions among 4 ANN models fed with different feature sets, we observed that ANN model trained with texture-GLCM had significantly better performance than that trained with texture-LBP and attenuation features (p < 0.001 and p = 0.006, respectively). Similar performance (texture-GCLM vs. all features, p = 1.000) was observed when training the ANN model with all features in comparison with the models trained with texture-LBP and attenuation features (both p < 0.001). No significant difference of AUC distribution was observed between the models trained with texture-LBP and attenuation features (p = 0.177).

### Discriminative Efficacy of Experienced Radiologists

In the discrimination between adenocarcinoma and NCH by two experienced radiologists, the sensitivity, specificity, and accuracy were 87.76% (43/49), 71.43% (30/42), and 80.2% (73/91).

## Discussion

Radiomics integrating the high-dimension features of CT data has its limitless potential ability to identify the attenuation distribution, which is unavailable to human visual resolution. Through our practice of imaging quantification, our major findings were as follows. First, NCH had a more heterogeneous internal distribution of attenuations than adenocarcinoma, which probably indicated the complex components of organization inside the NCH. Then, as we hypothesized, by implementing the imaging features into ANN classifier, NCH could be well discriminated from lung adenocarcinoma, which was obviously outperforming the experienced radiologists in diagnosing NCH (ANN classifier trained with texture-GLCM vs. experienced radiologists, 83.20% vs. 71.43%).

The heterogeneities of imaging features mainly depend on the differences of interrelationships between voxels, which could reflect the intra-tumoral heterogeneity (6, 7). Putting insight into the biological behavior of different tumors, a majority of evidences supported that the intra-tumoral heterogeneity generally results from the different tumor growth (21), internal necrosis (22), and complex organizations. Relative to adenocarcinoma being the most common malignant tumor in lung, NCH manifests a less progressive biological behavior indicating slower growth rate and less internal necrosis but larger heterogeneity of tissue organizations including fibrous connective tissue and the cartilage, fat in different proportions (23). Currently, we observed a significantly higher averaged contrast, cluster prominence, cluster shade, energy, and entropy in the NCH compared to the adenocarcinoma. All these features increase with an amplification of heterogeneity of attenuation distribution in NCH. Nevertheless, Dennie et al. reported that malignant tumors in the lung had more complex and inhomogeneous internal structure compared to a benign lesion (granulomatous nodule) quantified by texture analysis (24). The key reason for this inconsistency was that the benign lesions both studies included were with obviously different histological organizations. Indeed, NCH owns greatly complex organizations but is undetectable to human visual resolution. Therefore, the quantitative imaging features would be helpful to identify these differences. However, it may be suggestive that, as imaging quantification is widely used previously (6, 7), the heterogeneity is not always indicative of malignant lesions; the internal complex organization should be also taken into consideration.

Because those microscopic alterations (texture features) are imperceptible to human sight for its lacking of the diagnostic hallmark (calcification), patients with NCH are susceptible to accept invasive examinations including surgery resection. Therefore, it was significant for us to test the capacity of imaging quantification in discriminating NCH from adenocarcinoma, which is of great importance in future clinical application. Here, we confirmed that texture-GLCM analysis showed the highest efficacy to discriminate the lesions among the three kinds of features, which was demonstrated to outperform the invited experienced radiologists. Specifically, the performance of ANN classifier trained with texture-GLCM features to correctly diagnose NCH patients was significantly better than the radiologists (83.20% vs. 71.43%), while they had comparable ability to diagnose adenocarcinoma patients (87.22% vs. 87.87%). Taken together, in future clinical practice, if it is difficult to discriminate the lesions while NCH and adenocarcinoma are both suspected, such a machine-learning model trained with a texture-GLCM feature would contribute to identifying the internal distribution of attenuations and provide evidence for the discrimination.

There were some limitations in this study. First, though we collected the pathologically confirmed NCH with a distance of 5 years in our institute, the sample size here was relatively small, which made it difficult to perform external validation. Therefore, studies with larger sample size (e.g., multi-center database) would be expected to further facilitate the clinical translation. Second, the raw images were collected from different CT scanners. The existence of variability in image acquisition may influence the results, but that may not be evitable in the clinical practice. Third, the ROI segmentation was performed manually, which might be affected by the observers’ subjective bias. Nevertheless, for solid lesions, manual segmentation is stable rather than ground-glass lesions, and the DICE Similarity coefficients demonstrated an excellent inter-observer agreement in the segmentations. Fourth, the lack of other benign lesions consisting of single tissue component as another reference may limit future application; therefore, the alterations of radiomics features among multiple kinds of lesions should be explored in the future.

## Conclusions

Quantifying imaging features is a potentially useful tool for clinical diagnosis. Our study demonstrated that NCH has a heterogeneous distribution of attenuations probably reflecting its complex organizations. Based on this property, imaging quantification could improve the discrimination of NCH from adenocarcinoma.

## Data Availability Statement

All datasets presented in this study are included in the article/supplementary material.

## Ethics Statement

The studies involving human participants were reviewed and approved by The Medical Ethics Committee of the Second Affiliated hospital School of Medicine Zhejiang University. Written informed consent for participation was not required for this study in accordance with the national legislation and the institutional requirements.

## Author Contributions

All of the coauthors listed meet the criteria for authorship. XG was involved with study concept and design, acquisition of data, analysis and interpretation of data, and drafting/revising the manuscript. SW was involved with study design, data analysis, and interpretation. PK and HL were involved with acquisition of data, analysis, and interpretation of data. MZ was involved with data analysis and manuscript revision. DQ and XX were responsible for data interpretation, manuscript revision, obtaining funding, and study supervision. All authors contributed to the article and approved the submitted version.

## Funding

This work was supported by the National Key Research and Development Program of China (Grant No. 2017YFC0113400) and the Medical Health Science and Technology Project of Zhejiang Provincial Health Commission (No.2017198897).

## Conflict of Interest

The authors declare that the research was conducted in the absence of any commercial or financial relationships that could be construed as a potential conflict of interest.
